# Clinical outcomes of stereotactic body radiotherapy for stage I non-small cell lung cancer using different doses depending on tumor size

**DOI:** 10.1186/1748-717X-5-81

**Published:** 2010-09-17

**Authors:** Fumiya Baba, Yuta Shibamoto, Hiroyuki Ogino, Rumi Murata, Chikao Sugie, Hiromitsu Iwata, Shinya Otsuka, Katsura Kosaki, Aiko Nagai, Taro Murai, Akifumi Miyakawa

**Affiliations:** 1 Department of Radiology, Nagoya City University Graduate School of Medical Sciences, Nagoya, Japan; 2 Department of Radiology, Social Insurance Chukyo Hospital, Nagoya, Japan

## Abstract

**Background:**

The treatment schedules for stereotactic body radiotherapy (SBRT) for lung cancer vary from institution to institution. Several reports have indicated that stage IB patients had worse outcomes than stage IA patients when the same dose was used. We evaluated the clinical outcomes of SBRT for stage I non-small cell lung cancer (NSCLC) treated with different doses depending on tumor diameter.

**Methods:**

Between February 2004 and November 2008, 124 patients with stage I NSCLC underwent SBRT. Total doses of 44, 48, and 52 Gy were administered for tumors with a longest diameter of less than 1.5 cm, 1.5-3 cm, and larger than 3 cm, respectively. All doses were given in 4 fractions.

**Results:**

For all 124 patients, overall survival was 71%, cause-specific survival was 87%, progression-free survival was 60%, and local control was 80%, at 3 years. The 3-year overall survival was 79% for 85 stage IA patients treated with 48 Gy and 56% for 37 stage IB patients treated with 52 Gy (*p *= 0.05). At 3 years, cause-specific survival was 91% for the former group and 79% for the latter (*p *= 0.18), and progression-free survival was 62% versus 54% (*p *= 0.30). The 3-year local control rate was 81% versus 74% (*p *= 0.35). The cumulative incidence of grade 2 or 3 radiation pneumonitis was 11% in stage IA patients and 30% in stage IB patients (*p *= 0.02).

**Conclusions:**

There was no difference in local control between stage IA and IB tumors despite the difference in tumor size. The benefit of increasing the SBRT dose for larger tumors should be investigated further.

## Background

Stereotactic body radiotherapy (SBRT) for lung tumors was introduced in the mid 1990s [1], and it has been performed in many institutions as a new treatment modality for stage I primary lung cancer and oligometastatic lung cancer. Promising clinical results have been reported despite the use of various treatment protocols [2-9]. According to a recently published survey of SBRT in Japan, the treatment techniques and schedules applied for SBRT for lung cancer varied greatly from institution to institution [10]. The most frequently used schedule was 48 Gy in 4 fractions for both stage IA and IB primary lung cancer and metastatic lung cancer.

As a result, it was found that the outcomes of stage IB patients were worse than those of stage IA patients at the same dose [3-5], which suggests that SBRT doses should be adjusted according to tumor size. We have performed SBRT for lung tumors since 2004 and changed the prescribed dose depending on tumor diameter. In this study, we report the clinical outcomes of SBRT performed with our prospective hypothesis-driven protocol.

## Methods

### Eligibility Criteria

The eligibility criteria were as follows: histologically-confirmed non-small cell lung cancer (NSCLC) diagnosed as T1N0M0 or T2N0M0 stage according to the International Union Against Cancer 1997 system by CT of the chest and upper abdomen, bone scintigraphy, and brain magnetic resonance imaging and a World Health Organization performance status ≤ 2. When 18-fluoro-deoxyglucose-positron emission tomography (FDG-PET) was performed, bone scintigraphy was omitted. Even when the diagnosis of NSCLC could not be confirmed with transbronchial lung biopsy or CT-guided biopsy, such cases were included in the study if FDG-PET findings were positive and the tumor increased in size during the observation period. No restrictions were imposed with regard to the tumor location. Any patients who had undergone prior therapy were excluded. All patients consented to the treatment after they had been informed of the method and rationale of the study.

### Patient Characteristics

Between February 2004 and November 2008, 124 patients underwent SBRT for NSCLC. Eighty-four were men and 40 were women. The age at SBRT ranged from 26 to 89 years, with a median of 77 years. The tumor diameter ranged from 12 to 55 mm with a median of 27 mm. In 10 patients, NSCLC could not be histologically proven. The patient characteristics are summarized in Table 1.

**Table 1:** Patient characteristics

		Stage IA		Stage IB
			
Prescribed dose (in 4 fractions)	All	44 Gy	48 Gy	52 Gy
				
Patient number	124	2	85	37
Age (years)				
Range (median)	29-89 (77)	67, 70	58-87 (77)	29-89 (78)
Gender				
Male	84	1	54	29
Female	40	1	31	8
Performance status				
0	65	2	47	16
1	48	0	31	17
2	11	0	7	4
Tumor size (mm)				
Range (median)	12-55 (27)	12, 14	15-34 (24)	31-55 (35)
Operability				
Operable	40	2	27	11
Non-operable	84	0	58	26
Histology				
Adenocarcinoma	66	2	46	18
Squamous cell carcinoma	35	0	19	16
Unclassified NSCLC	13	0	10	3
Unproven	10	0	10	0
Tumor location				
Central	29	0	18	11
Peripheral	95	2	67	26

Abbreviations: NSCLC = non-small cell lung cancer.

### Treatment methods

Our methods for immobilization and treatment planning were described in detail previously [11]. We used the BodyFIX system (Medical Intelligence, Schwabmuenchen, Germany) for patient immobilization. CT images for treatment planning were obtained under normal breathing, and with breath holding during the expiratory and inspiratory phases. The clinical target volume (CTV) was defined as the visible gross tumor volume (GTV). The CTV on CT during the 3 phases were superimposed on a 3-dimensional radiation treatment planning system (Eclipse Version 7.5.14.3, Varian Medical Systems, Palo Alto, California, USA) to represent the internal target volume (ITV). We defined the planning target volume (PTV) margin for the ITV as 5 mm in the lateral and anteroposterior directions and 10 mm in the craniocaudal direction. Three coplanar and 4 noncoplanar static ports were used. SBRT was delivered by a linear accelerator (CLINAC 23EX, Varian Medical Systems, Palo Alto, California, USA) with 6-MV photons. The treatment was performed twice a week. The median treatment period was 11 days.

### Prescription dose

The dose was prescribed according to the tumor diameter. The planned dose was 44 Gy in 4 fractions for tumors with a maximum diameter of less than 1.5 cm, 48 Gy in 4 fractions for tumors with a maximum diameter of 1.5-3 cm, and 52 Gy in 4 fractions for those with a maximum diameter larger than 3 cm. Assuming an α/β ratio of 10 Gy, the biological effective dose (BED) was 92 Gy for the 44-Gy schedule, 106 Gy for the 48-Gy schedule, and 120 Gy for the 52-Gy schedule. However, the BED must be cautiously used in these dose-fractionation ranges [12]. Pencil beam convolution with Batho power law correction of the Eclipse system was used as the dose calculation algorithm. The prescribed dose represented that delivered to the isocenter, and it was ensured that 95% of the PTV received at least 80% of the prescribed isocenter dose. Dose constraints were set for the spinal cord, and only one of the beams was allowed to pass the spinal cord.

### Evaluation

For follow-up after SBRT, chest CT was performed at 2-month intervals until 6th months, and every 2 to 4 months thereafter. FDG-PET was performed whenever necessary. Local recurrence was suspected when enlargement of a consolidated fibrotic mass was detected on CT images without signs of inflammation and was diagnosed by high uptake on FDG-PET (standardized uptake value > 5) and/or biopsy. Local recurrence was confirmed by biopsy in 2 patients. Toxicity was evaluated using the Common Terminology Criteria for Adverse Events Version 3. Grade 2 radiation pneumonitis was defined as symptomatic but not interfering with activities of daily life.

### Statistical Analysis

The unpaired t-test or the Mann-Whitney U test was used to compare the characteristics of the patients. Survival rates and cumulative incidences of complications were calculated by the Kaplan-Meier method from the start of SBRT. The log-rank test was used to compare the control and survival rates between the subsets. Statistical analysis was carried out using StatView software version 5.0 (SAS Institute, Cary, NC).

## Results

### Survival

Among 124 NSCLC patients treated with SBRT, 87 had stage IA and 37 had stage IB disease. Two stage IA patients with tumors of less than 1.5 cm in diameter were treated with 44 Gy in 4 fractions, and 85 patients with larger T1 tumors were treated with 48 Gy in 4 fractions. All 37 stage IB patients were treated with 52 Gy in 4 fractions. There were no significant differences in the distribution of age (*p *= 0.95), gender (*p *= 0.11), PS (*p *= 0.26), operability (*p *= 0.82), histology (*p *= 0.71), or tumor location (*p *= 0.31) between the 85 stage IA patients treated with 48 Gy in 4 fractions and the 37 stage IB patients. The median follow-up period for living patients was 26 months (range: 7 to 66 months). Local recurrence developed in 18 patients (11 among the stage IA patients and 7 among the stage IB patients). Regional lymph node recurrence occurred in 19 patients (10 among the stage IA patients and 9 among the stage IB patients). Distant metastases appeared in 25 patients (16 among the stage IA patients and 9 among the stage IB patients).

**Figure 1:**
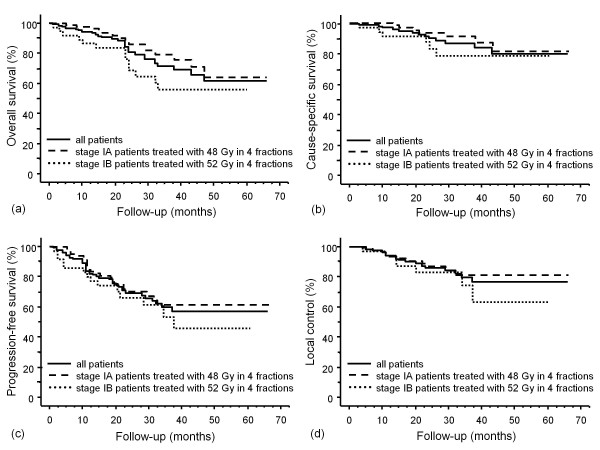
**Curves for (a) overall survival, (b) cause-specific survival, (c) progression-free survival, and (d) local control in stage I NSCLC patients**. Solid line, all patients (n = 124); dashed line, stage IA patients treated with 48 Gy in 4 fractions (n = 85); and dotted line, stage IB patients treated with 52 Gy in 4 fractions (n = 37).

For all 124 patients, the overall survival (OAS) rate was 71%, the cause-specific survival (CSS) rate was 87%, and the progression-free survival (PFS) rate was 60% at 3 years (Figure 1). The 3-year OAS was 79% for the 85 stage IA patients treated with 48 Gy in 4 fractions and 56% for the 37 stage IB patients (*p *= 0.05). The 3-year CSS was 91% for the former group and 79% for the latter (*p *= 0.18). The 3-year PFS was 62% versus 54% (*p *= 0.30). The 3-year local control rate was 80% for all patients, and it was 81% for the stage IA patients treated with 48 Gy and 74% for the stage IB patients, with no significant difference between them (*p *= 0.35). Two stage IA patients treated with 44 Gy in 4 fractions were alive without recurrence at 21 and 14 months, respectively.

Treatment outcomes were also analyzed with respect to the tumor location [13]. The 3-year OAS was 72% for patients with tumors in the central (perihilar or central mediastinal) region and 71% for those with tumors in the peripheral region (*p *= 0.63). The 3-year CSS was 82% for patients with central tumors and 89% for patients with peripheral tumors (*p *= 0.63). The 3-year PFS was 52% versus 62% (*p *= 0.79), and the 3-year local control was 66% versus 83% (*p *= 0.33).

### Toxicities

Grade 1, 2, and 3 radiation pneumonitis was observed in 66, 17, and 2 patients, respectively. At 3 years, the cumulative incidence of grade 2 or 3 pneumonitis was 16%, and it was 11% for stage IA patients treated with 48 Gy in 4 fractions and 30% for stage IB patients treated with 52 Gy in 4 fractions (*p *= 0.02). Other adverse events were as follows: grade 2 esophagitis was seen in 3 patients, grade 1 and 3 pleural effusion were detected in 23 and 1 patient(s), respectively; grade 1 atelectasis was found in 6 patients; grade 1 pneumothorax was detected in 3 patients; grade 1 and 2 dermatitis were observed in 7 and 6 patients, respectively; grade 1 and 2 rib fractures were seen in 7 and 1 patient(s), respectively; grade 1 soft tissue swelling was detected in 6 patients; and grade 2 cardiac muscle damage and effusion were detected in 1 patient each. At 3 years, the cumulative incidence of grade 2 or 3 radiation pneumonitis was 25% in patients with central tumors and 13% in patients with peripheral tumors (*p *= 0.11).

## Discussion

Following the excellent clinical outcomes reported by Nagata et al. [2], the most frequently used schedule for SBRT for NSCLC in Japan has been 48 Gy in 4 fractions for both stage IA and IB tumors [10]. However, other investigators reported worse outcomes in stage IB patients when the same fractionation schedule was used [3-5]. Their protocols are summarized in Table 2. Onimaru et al. [3] reported 3-year CSS rates of 88% and 50% for stage IA and IB patients, respectively. Significant differences were found in OAS, CSS, and local control rates between stage IA and IB tumors. Koto et al. [4] also showed that the 3-year local control rate was 78% and 40% for stage IA and IB, respectively. Baumann et al. [5] reported that the estimated risk of all failures was increased in stage IB patients compared with stage IA patients. Onishi et al. [14] reported the results of a multi-institutional study. The irradiation schedules of participating institutions involved total doses of 30 to 84 Gy (at the isocenter) delivered in 1 to 14 fractions. Although the treatment protocols varied greatly, the 5-year OAS rate in operable groups receiving a sufficient dose was better in stage IA than in stage IB patients. In general, greater doses are needed to control larger tumors in conventional radiotherapy [15-17]. The above-mentioned results suggest that the control rates for stage IB tumors should be lower than those for stage IA tumors at the same dose. On the other hand, smaller doses could be sufficient for controlling smaller tumors. Takeda et al. [6] also administered the same dose and achieved favorable outcomes in both stage IA and IB tumors. This suggests that if a sufficient dose is administered in a certain number of fractions, stage IB tumors can be controlled as well as stage IA tumors. One study by Fakris et al. [7] prescribed a greater dose for stage IB tumors than for stage IA (Table 2), and they reported no significant difference in median survival or 3-year CSS between stages IA and IB. We also prescribed a greater dose for stage IB tumors. The CSS, PFS, and local control rates for stage IB patients were not significantly different from those for stage IA patients.

**Table 2:** Protocols for stage IA and IB NSCLC

First author (Ref)	Prescribed dose (Gy/fraction)	Reference point	Isocenter dose (Gy/fraction)	Calculation algorithm/inhomogeneity correction
Nagata (2)	48/4	isocenter	48/4	PBC /yes
Onimaru (3)	40 or 48/4	isocenter	40 or 48/4	Clarkson or superposition /yes
Koto (4)	45/3 or 60/8	isocenter	45/3 or 60/8	BPL /yes
Baumann (5)	45/3	67% at PTV periphery	67.2/3	PBC /yes
Takeda (6)	50/5	100% at PTV periphery	62.5/5	MG superposition /yes
Fakris (7)	60/3* 66/3**	80% at least 95% of PTV	at least 79.4/3* at least 86.8/3**	unspecified /no
Our study	48/4* 52/4**	isocenter	48/4* 52/4**	PBC /yes

* = for stage IA, ** = for stage IB.

Abbreviations: PBC = Pencil beam convolution; BPL = Batho power law; MG = Multigrid.

The local control rates in our study do not seem to be high enough compared with the rates in other recent reports [5-7,18]. In particular, a recent Radiation Therapy Oncology Group study obtained a 3-year local control rate of 97.6% using 54 Gy in 3 fractions delivered to the periphery of the PTV [18]. Guckenberger et al. [19] indicated a dose-response relationship for local control in pulmonary SBRT. Our doses might have been insufficient for local control in a certain proportion of patients. We delivered the dose to the isocenter using Pencil beam convolution with Batho power law correction, and we ensured that 95% of the PTV received at least 80% of the prescribed dose. However, the dose distribution at the PTV periphery might have been insufficient [6,20]. We think it is necessary to use a more accurate inhomogeneity correction algorithm to improve dose conformality. It might also be argued that only using 7 beams resulted in inferior dose conformality compared to using more beams. However, in our analyses before this study, 7 beams were considered acceptable. Indeed, the mean V20 (volume of lung minus GTV receiving ≥ 20 Gy: 6.7% ± 2.9% [SD]) and the mean lung dose (MLD: 4.5 ± 1.5 Gy [SD]) for all patients in the present study were not inferior to those reported by other investigators [21-23].

Since greater doses were prescribed to a larger PTV, the normal tissues around the PTV absorbed greater doses, which may have increased toxicities in normal organs. Radiation pneumonitis is the most significant dose-related toxicity. Some dose-volume parameters such as the V20 and MLD are reported to correlate with radiation pneumonitis [24,25]. Takeda et al. [21] reported a linear correlation between tumor diameter and V20 in SBRT. The mean PTV (± SD) of tumors irradiated with 48 Gy and 52 Gy in 4 fractions was 45 ± 21 cm^3 ^and 78 ± 25 cm^3^, respectively (*p *< 0.0001). So, the PTV of stage IB tumors was significantly larger than that of stage IA tumors. The V 20 was 5.9% ± 2.3% for the 48-Gy group and 8.4% ± 3.5% for the 52-Gy group (*p *< 0.0001), and the MLD were 4.1 ± 1.2 Gy and 5.4 ± 1.8 Gy, respectively (*p *< 0.0001). These data indicate that a greater dose was absorbed in the normal lung. This was considered to have caused the significantly higher cumulative incidence of grade 2 or 3 radiation pneumonitis in the stage IB patients treated with 52 Gy in 4 fractions compared with that of the stage IA patients treated with 48 Gy in 4 fractions in our study. A dose-response relationship for radiation-induced pneumonitis after SBRT has also been reported recently [22,23].

Various dose fractionation schedules have been used in SBRT for lung cancers [26], and optimum schedules have been sought. A future topic for study is dose escalation, and another is to combine SBRT with chemotherapy to improve outcomes. The Japan Clinical Oncology Group is conducting a dose escalation study for stage IB NSCLC. Stage IB tumors are not only difficult to control, but are also associated with more occult distant metastases than stage IA tumors. So, combined chemotherapy could be effective. Chen et al. [27] demonstrated that SBRT followed by adjuvant chemotherapy improved OAS. In our protocol, the toxicities associated with stage IA tumors were mild, so it seems possible that dose escalation for stage IA tumors would improve local control and survival rates. Most patients with stage IB tumors are elderly or medically inoperable, and dose escalation with more conformal approaches should be investigated for such patients. On the other hand, considering the relatively high pulmonary toxicities observed in our stage IB patients, combined chemotherapy might be a future strategy for improving the survival of medically operable stage IB patients.

## Conclusions

A protocol involving 44, 48, or 52 Gy being delivered in 4 fractions to the isocenter was feasible for patients with stage IA or IB NSCLC. There was no difference in local control between stage IA and IB tumors despite the difference in tumor size. The benefit of increasing the doses for larger tumors should be investigated further.

## Competing interests

The authors declare that they have no competing interests.

## Authors' contributions

FB carried out the study and drafted the manuscript. YS designed the study and gave final approval for publication. HO participated in the design of the study and helped to perform the statistical analyses. RM and CS participated in the analysis and the data interpretation. HI, SO, KK, and AN participated in the data acquisition and analysis. TM and AM contributed to the data acquisition. All authors have read and approved the final manuscript.
